# The Parzen Window method: In terms of two vectors and one matrix^☆^

**DOI:** 10.1016/j.patrec.2015.06.002

**Published:** 2015-10-01

**Authors:** Hamse Y. Mussa, John B.O. Mitchell, Avid M. Afzal

**Affiliations:** a EaStCHEM School of Chemistry and Biomedical Sciences Research Complex, University of St Andrews, North Haugh, St Andrews KY16 9ST, Scotland, UK; b Centre for Molecular Informatics, Department of Chemistry, University of Cambridge, Lensfield Road, Cambridge CB2 1EW, England, UK

**Keywords:** Probability density function, Kernel functions, Parzen Window

## Abstract

Pattern classification methods assign an object to one of several predefined classes/categories based on features extracted from observed attributes of the object (pattern). When *L* discriminatory features for the pattern can be accurately determined, the pattern classification problem presents no difficulty. However, precise identification of the relevant features for a classification algorithm (classifier) to be able to categorize real world patterns without errors is generally infeasible. In this case, the pattern classification problem is often cast as devising a classifier that minimizes the misclassification rate. One way of doing this is to consider both the pattern attributes and its class label as random variables, estimate the posterior class probabilities for a given pattern and then assign the pattern to the class/category for which the posterior class probability value estimated is maximum. More often than not, the form of the posterior class probabilities is unknown.

The so-called Parzen Window approach is widely employed to estimate class-conditional probability (class-specific probability) densities for a given pattern. These probability densities can then be utilized to estimate the appropriate posterior class probabilities for that pattern. However, the Parzen Window scheme can become computationally impractical when the size of the training dataset is in the tens of thousands and *L* is also large (a few hundred or more). Over the years, various schemes have been suggested to ameliorate the computational drawback of the Parzen Window approach, but the problem still remains outstanding and unresolved.

In this paper, we revisit the Parzen Window technique and introduce a novel approach that may circumvent the aforementioned computational bottleneck. The current paper presents the mathematical aspect of our idea. Practical realizations of the proposed scheme will be given elsewhere.

## Highlights

• We revisit the Parzen Window approach widely employed in pattern recognition.
• The Parzen Window approach can suffer from a severe computational bottleneck.
• This manuscript introduces a new scheme to ameliorate this computational drawback.

## 1 Introduction

In mathematical pattern recognition, the problem of pattern classification entails assigning an object – based on a number of specific features of the object – to one of a finite set of predefined classes/categories *ω_j_*, where *j =*1, 2,…, *J*, with *J* being the number of classes/categories of interest. Typically the object (or simply the pattern) is represented by an *L*-dimensional vector **x** whose elements, $(x_{1},x_{2},\ldots ,x_{L}),$ are values assumed to contain the appropriate information about the specific pattern features utilized to accurately classify the pattern represented by **x**.

When *L* discriminatory features for a pattern can be determined accurately, the pattern classification problem presents no difficulty: it reduces to a simple look-up table scheme. However, identifying the relevant features to classify realistic patterns without classification errors is generally impossible. Thus, the pattern classification problem is often cast as the task of finding a classifier that minimizes the misclassification rate [1]. One popular way of achieving this objective is to treat both the pattern vector **x** and the class label *ω_j_* as random variables. In this case, the posterior class probabilities *p*(*ω_j_*|**x**) for a given pattern **x** is computed; then pattern **x** is assigned to the class, for which the *p*(*ω_j_*|**x**) value is maximum [1–6]. (In the last step it is being assumed that all misclassification errors are equally bad [1,3,4].)

However, in practice, the form of the function *p*(*ω_j_*|**x**) is unknown; instead, *N* patterns **x**_*i*_ and their corresponding correct class labels $y_{i}\in \left\{\omega_{1},\omega_{2},\ldots ,\omega_{J}\right\}$ – i.e., $D={\left\{\left(x_{i},y_{i}\right)\right\}}_{i=1}^{N},$ assumed to constitute a representative dataset of the joint probability density function *p*(*ω_j_*, **x**) for *ω_j_* and **x** – are usually available. It is from these prototype patterns **x**_*i*_ and their corresponding class labels *y_i_* that one tries to estimate *p*(*ω_j_*|**x**).

According to basic probability rules [1–7],

(1) $$p\left(\omega_{j}|x\right)p\left(x\right)=p\left(\omega_{j},x\right)=p\left(x|\omega_{j}\right)p\left(\omega_{j}\right)$$

These rules allow one to modularize the estimation problem and estimate *p*(*ω_j_*|**x**) (and, of course, *p*(**x**)) in terms of *p*(**x**|*ω_j_*) and *p*(*ω_j_*):

(2) $$p\left(\omega_{j}|x\right)=\frac{p\left(x|\omega_{j}\right)p\left(\omega_{j}\right)}{p(x)},$$

whereby we may have a better chance of being able to estimate *p*(**x**|*ω_j_*) and *p*(*ω_j_*) from D than estimating *p*(*ω_j_*|**x**) directly from D. In the denominator, $p\left(x\right)=\sum_{j=1}^{J}p\left(x|\omega_{j}\right)p\left(\omega_{j}\right)$.

In the Bayesian statistics framework, *p*(*ω_j_*) is referred to as the class prior probability, which is the probability that a member of class *ω_j_* will occur. The function *p*(**x**|*ω_j_*) is called the class-conditional probability density function, i.e. the probability density of observing pattern **x** given that **x** is a member of class *ω_j_*. The denominator term on the right hand side of Eq. 2 is often called the “evidence” or “marginal likelihood”. For the purpose of this paper we can afford to simply view this term as a normalization factor.

If there is evidence that the number of prototype patterns per class is an indication of the importance of that class, then a sensible approximation of *p*(*ω_j_*) can be

(3) $$p\left(\omega_{j}\right)=\frac{\sum_{i=1}^{N}\nu_{j}^{i}}{N},$$

where $\nu_{j}^{i}=1$ if the *i*th prototype **x**_*i*_ belongs to class *ω_j_*, otherwise $\nu_{j}^{i}=0$; and *N* is as described before. Nonetheless, *p*(*ω_j_*) is typically assumed to be uniform, i.e., $p\left(\omega_{j}\right)=\frac{1}{J},$ where *J* is as defined before.

Estimating *p*(**x**|*ω_j_*) from D is not straightforward [1–5]. In the last half-century, a plethora of methods have been proposed for estimating *p*(**x**|*ω_j_*) based on D, the so-called training set. There are ample excellent reviews and text books on this topic; for example, the two books – one by Hand [4] and the other by Murphy [8] – give adequate and accessible descriptions of the bulk of these approaches devised in recent (and not so recent) years.

In this paper we are concerned with one particular approach that is widely thought to be apropos to the task of estimating *p*(**x**|*ω_j_*) from a representative training dataset: the so-called Parzen Window method [1,2,4,9,10], also known as Parzen estimator, Potential function technique [10], to name but a few.

In the preceding discussion and in the rest of the paper, the terms “class”, “label”, “class label” and “category” are employed interchangeably. For notational simplicity we use **x, x**_*i*_ and *ω_j_* both as random variables and their realizations. Furthermore we follow (in line with the current trend in machine learning and statistics) the convenient – but not necessarily correct – practice of using the term “density” for both a discrete random variable’s probability function and for the probability function of a continuous random variable. An implicit assumption being made throughout the paper is that all spaces, matrices, vectors, functions and variables (discrete or not), *etc*., are real.

## 2 Literature review

The Parzen Window approach can approximate *p*(**x**|*ω_j_*) by a simple formula [1,2,10]:

(4) $$\hat{p}\left(x|\omega_{j}\right)=\frac{1}{\sum_{i=1}^{N}\nu_{j}^{i}}\sum_{i=1}^{N}K\left(x,x_{i};\lambda \right)\nu_{j}^{i},$$

where $x_{i}\in D$ denotes prototype patterns and $\nu_{j}^{i}$ is as defined before. *K*(**x, x**_*i*_; *λ*) – commonly known as the kernel function – is a two-variable function with specific properties, which are abundantly covered in the statistical pattern recognition literature [1,2,9,10]. At any rate, it might be helpful to think of *K*(**x, x**_*i*_; *λ*) as a measure of similarity returning how similar patterns **x** and **x**_*i*_ are, *λ* being a tunable (smoothing) parameter. In other words, the kernel function peaks at **x** = **x**_*i*_ and decays away elsewhere; the *λ* parameter, *inter alia*, has an important role in determining the rate of the decay.

Eq. 4 indicates that $\hat{p}\left(x|\omega_{j}\right)$ is formed from the superposition of kernel function *K*(**x**_*i*_, **x**; *λ*) values at the given prototype patterns **x**_*i*_ for class *ω_j_*. The Parzen Window method is powerful in the sense that, with enough representative data points (prototypes/references), its estimate of the class conditional probability density converges to *p*(**x**|*ω_j_*) (see Ref. [1], Chapter 4). Although Eq. 4 is conceptually simple and capable of providing a good estimate of *p*(**x**|*ω_j_*), it can computationally suffer from the requirements that all the prototypes/references **x**_*i*_ for class *ω_j_* must be retained in main-memory to compute $\hat{p}\left(x|\omega_{j}\right),$ the estimate of *p*(**x**|*ω_j_*). Furthermore, considerable CPU-time may be required each time this method is used to estimate *p*(**x**|*ω_j_*) to classify a novel pattern. The fact that the size of the reference pattern vectors **x**_*i*_ can be easily in the hundreds (or more) may exacerbate the main-memory and CPU-time requirements.

Over the years, various schemes have been developed to address the computational drawback of this otherwise elegant and powerful method. For example, one of these schemes entails – see Ref. [1] (Chapter 4), Ref. [4] (Chapter 2) and Ref. [10] (Chapters 6) for detailed technical and practical discussions – expressing the kernel function as a finite series expansion

(5) $$K\left(x,x_{i};\lambda \right)=\sum_{m=1}^{M}\phi_{m}\left(x\right)\phi_{m}\left(x_{i}\right),$$

which renders the right hand side of Eq. 4

(6) $$\frac{1}{\sum_{i=1}^{N}\nu_{j}^{i}}\sum_{i=1}^{N}K\left(x,x_{i};\lambda \right)\nu_{j}^{i}=\frac{1}{\sum_{i=1}^{N}\nu_{j}^{i}}\sum_{i=1}^{N}\nu_{j}^{i}\sum_{m=1}^{M}\phi_{m}\left(x\right)\phi_{m}\left(x_{i}\right),$$

with ${\left\{\phi_{m}\right\}}_{m=1}^{M}$ being appropriate basis functions (not necessarily polynomials) defined in the feature space in which the pattern vectors **x** and **x**_*i*_ reside.

From Eqs. 4 and 6, we have

(7) $$\hat{p}\left(x|\omega_{j}\right)=\sum_{m=1}^{M}c_{m_{\omega_{j}}}\phi_{m}\left(x\right),$$

where

$$c_{m_{\omega_{j}}}=\frac{1}{\sum_{i=1}^{N}\nu_{j}^{i}}\sum_{i=1}^{N}\nu_{j}^{i}\phi_{m}\left(x_{i}\right),$$

with $\nu_{j}^{i}=1$ if pattern **x**_*i*_ belongs to class *ω_j_*, otherwise $\nu_{j}^{i}=0$.

This scheme certainly removes the reference patterns’ storage problem. However, it can create a computational problem of its own, in particular when both *M* and *L* are large, which is often the case in real world classification problems. Computing *M* basis functions of *L* variables to classify a new pattern **x** is not a trivial computational task [1–4,10–12].

Another approach – albeit a particular case of the scheme above – is that proposed by Specht [13]. It was based on a Taylor series expansion of *ρ*(**x, x**_*i*_; *λ*) (see Eq. 8), such that an *r*th order polynomial in *L* variables was required to estimate and store (L+rL) terms to approximate $\hat{p}\left(x|\omega_{j}\right)$ [10,13,14]. For short but “insightful” descriptions of the relationship between an appropriate value of *r* and the smoothing parameter *λ*, see Ref. [1] (Chapter 4) and Ref. [14] (Chapter 4). In principle, Specht’s scheme has a strong appeal of simplicity providing the number of terms required in the Taylor series can be held to a practical limit. Unfortunately, both *r* and *L* can be large in current realistic classification problems [1,4,10].

Despite these (and many other) efforts, to the best of our knowledge, the computational bottleneck that the Parzen Window method encounters, when *N* and *L* are large, remains an unresolved issue.

Thus, the motivation for this paper is to introduce yet another scheme that might be able to circumvent the aforementioned computational bottleneck problem, while retaining the estimation power and conceptual simplicity of the Parzen Window method. This work is confined to Parzen Window based approaches, in which the kernel function is – or can be expressed – in the form

(8) $$K\left(x,x_{i};\lambda \right)=Af\left(x;\lambda \right)\rho \left(x,x_{i};\lambda \right)f\left(x_{i};\lambda \right),$$

where *A* > 0; *f*(**x**; *λ*) and *f*(**x**_*i*_; *λ*) are any real functions defined in the feature space; *ρ*(**x, x**_*i*_; *λ*) is a polynomial in **x** and **x**_*i*_; and *λ* is as defined before. The kernel functions that are or can be written in the form above are ubiquitous nowadays in data analysis [4,6,8]. They have most often been successfully applied to discrete data; for this reason we decided to confine attention to the discrete case. For illustrative purposes, we focus on binary data, i.e., *x_l_* = 0 or 1 denoting absence or presence of feature *x_l_* in the pattern vector, respectively. That is to say, both **x** (test pattern vector) and **x**_*i*_ (reference/prototype pattern vector) reside in a binary feature space $X={\left\{0,1\right\}}^{L}$.

The extension of the proposed scheme to continuous data – i.e., $X=R^{L}$ – is straightforward.

One final, but important remark is that Specht’s approach and our proposed scheme are arguably similar in spirit. However there are crucial differences: unlike Specht’s formulation, our scheme does not estimate (L+rL) terms, it does not retain (L+rL) terms in main-memory, nor does the variable *r* feature in the final form of our algorithm – instead, in our case, only two *L*-dimensional vectors and one *L*-by-*L* matrix are required to retain in main-memory. The two vectors and the matrix can notably be highly sparse. We will briefly expound on this assertion shortly.

## 3 Proposed method and discrete Parzen Window approach

As a concrete example, we use the widely utilized kernel function (albeit in cheminformatics [15,16], and references therein) introduced by Aitchison and Aitken (AA–kernel) [17,18],

(9) $$K\left(x,x_{i};\lambda \right)=\lambda^{L}{\left(\frac{1-\lambda }{\lambda }\right)}^{d(x,x_{i})},$$

where 0.5 < *λ* < 1 and *d*(**x, x**_*i*_) denotes the number of components in which **x** and **x**_*i*_ disagree. This dissimilarity measure *d*(**x, x**_*i*_) can be conveniently expressed as [4]

(10) $$d\left(x,x_{i}\right)=x^{T}x-2x^{T}x_{i}+x_{i}^{T}x_{i}$$

In passing, the AA–kernel is basically a discrete analogue of an isotropic Gaussian kernel [17,18].

From Eqs. 9 and 10, and the fact that $e^{\ln w}=w,$ we have

(11) $$\begin{array}{ccc} K(x,x_{i};\lambda ) & = & \lambda^{L}e^{\ln\left(\frac{1-\lambda }{\lambda }\right)\left(x^{T}x+x_{i}^{T}x_{i}\right)}\times e^{-2\ln\left(\frac{1-\lambda }{\lambda }\right)x^{T}x_{i}}\\ & = & \lambda^{L}e^{-\alpha (x^{T}x+x_{i}^{T}x_{i})}\times e^{2\alpha x^{T}x_{i}}\\ & = & \lambda^{L}e^{-\alpha (x^{T}x)}\times e^{2\alpha x^{T}x_{i}}\times e^{-\alpha (x_{i}^{T}x_{i})}, \end{array}$$

where $\alpha =\ln(\frac{\lambda }{1-\lambda })$. The term $e^{2\alpha x^{T}x_{i}}$ can be written as

(12) $$\begin{array}{ccc} e^{2\alpha x^{T}x_{i}} & = & \sum_{r=0}^{\infty }\frac{{\left(2\alpha \right)}^{r}}{r!}{\left(x^{T}x_{i}\right)}^{r}=\sum_{r=0}^{\infty }\gamma_{r}{\left(x^{T}x_{i}\right)}^{r}, \end{array}$$

where *γ_r_* = $\frac{{\left(2\alpha \right)}^{r}}{r!}$.

Inserting Eq. 12 into Eq. 11 yields

(13) $$K\left(x,x_{i};\lambda \right)=\lambda^{L}e^{-\alpha (x^{T}x)}\left[\sum_{r=0}^{\infty }\gamma_{r}{\left(x^{T}x_{i}\right)}^{r}\right]e^{-\alpha (x_{i}^{T}x_{i})}$$

(*cf.* Eq. 8).

From Eqs. 13 and 4, we have

(14) $$\hat{p}\left(x|\omega_{j}\right)=\frac{\lambda^{L}e^{-\alpha (x^{T}x)}}{\sum_{i=1}^{N}\nu_{j}^{i}}\sum_{i=1}^{N}\nu_{j}^{i}e^{-\alpha (x_{i}^{T}x_{i})}\sum_{r=0}^{\infty }\gamma_{r}{\left(x^{T}x_{i}\right)}^{r}$$

Now we come to the main contribution of this paper: removing the requirement for retaining all the reference/prototype patterns for class *ω_j_* in main-memory to compute $\hat{p}\left(x|\omega_{j}\right)$ in order to estimate $\hat{p}\left(\omega_{j}|x\right)$ to classify a new pattern **x**. However, first we simplify the notation by defining these variables $N_{\omega_{j}},$ *a*, **z** and **z**′, which will be consistently used throughout, as follows: $N_{\omega_{j}}=\sum_{i=1}^{N}\nu_{j}^{i};\beta_{i}=e^{-\alpha (x_{i}^{T}x_{i})};a=\sum_{i=1}^{N_{\omega_{j}}}\beta_{i};z=\sum_{i=1}^{N_{\omega_{j}}}x_{i};$ and $z^{\prime }=\sum_{i=1}^{N_{\omega_{j}}}\beta_{i}x_{i},$ where $N_{\omega_{j}}$ refers to the number of patterns in the training dataset that belong to class *ω_j_; N, α* and $\nu_{j}^{i}$ are as described before. In our new notation, Eq. 14 becomes

(15) $$\begin{array}{ccc} \hat{p}\left(x|\omega_{j}\right) & = & B\left[\gamma_{0}\sum_{i=1}^{N_{\omega_{j}}}\beta_{i}+\sum_{r=1}^{\infty }\gamma_{r}\sum_{i=1}^{N_{\omega_{j}}}\beta_{i}{\left(x^{T}x_{i}\right)}^{r}\right]\\ & = & B\left[\gamma_{0}a+\sum_{r=1}^{\infty }\gamma_{r}\sum_{i=1}^{N_{\omega_{j}}}\left(x^{T}\left(\beta_{i}x_{i}\right)\right){\left(x^{T}x_{i}\right)}^{r-1}\right], \end{array}$$

where $B=\frac{\lambda^{L}e^{-\alpha (x^{T}x)}}{N_{\omega_{j}}}$.

The main contribution of the paper is formulating Eq. 15 in terms of *γ_r_* , *a*, **z, z**′ and an *L*-by-*L* matrix, **Q** which will be defined shortly. The task of this formulation basically amounts to expressing $\sum_{i=1}^{N_{\omega_{j}}}\left(x^{T}\left(\beta_{i}x_{i}\right)\right){\left(x^{T}x_{i}\right)}^{r-1}$ in terms of **z, z**′ and **Q**. In doing this, we hope to ameliorate the computational drawback of the Parzen Window method based on kernel functions in the form given in Eq. 8.

## 4 Results

When r=1, the task is trivial: $\sum_{i=1}^{N_{\omega_{j}}}\left(x^{T}\left(\beta_{i}x_{i}\right)\right){\left(x^{T}x_{i}\right)}^{r-1}$ reduces to

(16) $$\sum_{i=1}^{N_{\omega_{j}}}x^{T}\left(\beta_{i}x_{i}\right)=x^{T}z^{\prime }$$

where, by definition (see Section 3), $z^{\prime }=\sum_{i=1}^{N_{\omega_{j}}}\left(\beta_{i}x_{i}\right)$.

However, when *r* > 1, the task can be taxing. To this end, we make use of a simple – but useful – proposition (Proposition 1, whose proof is provided in Appendices A and B) to demonstrate that $\sum_{i=1}^{N_{\omega_{j}}}\left(x^{T}\left(\beta_{i}x_{i}\right)\right){\left(x^{T}x_{i}\right)}^{r-1}$ for *r* > 1 can be written as (see Eq. 19 in Appendix C)

(17) $$\sum_{i=1}^{N_{\omega_{j}}}\left(x^{T}\left(\beta_{i}x_{i}\right)\right){\left(x^{T}x_{i}\right)}^{r-1}=\left(x^{T}Qx\right){\left(x^{T}z\right)}^{r-2},$$

where **Q** is just an *L*-by-*L* matrix, (see Eq. 19).

Inserting Eqs. 16 and 17 into Eq. 15 results in

(18) $$\begin{array}{ccc} \hat{p}\left(x|\omega_{j}\right) & = & B\left[\gamma_{0}a+\gamma_{1}\left(x^{T}z^{\prime }\right)+\left(x^{T}Qx\right)\sum_{r=2}^{\infty }\gamma_{r}{\left(x^{T}z\right)}^{r-2}\right]\\ & = & B\left[\gamma_{0}a+\gamma_{1}\left(x^{T}z^{\prime }\right)+\left(\frac{4\alpha^{2}\left(e^{\mu }-\mu -1\right)}{\mu^{2}}\right)\left(x^{T}Qx\right)\right], \end{array}$$

where $B=\frac{\lambda^{L}e^{-\alpha (x^{T}x)}}{N_{\omega_{j}}}$; $\sum_{r=2}^{\infty }\gamma_{r}{\left(x^{T}z\right)}^{r-2}=\frac{4\alpha^{2}\left(e^{\mu }-\mu -1\right)}{\mu^{2}},$ with $\mu =2\alpha (x^{T}z),$ *γ_r_* = $\frac{{\left(2\alpha \right)}^{r}}{r!},$$\alpha =\ln(\frac{\lambda }{1-\lambda })$ and 0.5 < *λ* < 1

Evidently, Eq. 18 illustrates that it is not necessary to retain all reference/prototype patterns for a given class in main-memory to compute the value of $\hat{p}\left(x|\omega_{j}\right)$ for a test pattern **x**; instead, all that is required is an *L*-by-*L* matrix **Q** and two *L* size vectors (**z** and **z**′), which are computed once and then retained in main-memory. This was the objective we set out to achieve in this paper.

One final, but important, remark is that **z, z**′ and **Q** can be highly sparse in real world applications when *x_l_* ∈ {0, 1}, in particular if the value of *L* is large. The fundamental reason for this sparsity is that in a high-dimensional reference pattern vector **x**_*i*_ there is the potential of many of its components being zero – i.e., many of the features are very likely to be absent in **x**_*i*_.

In passing, if we are dealing with continuous data, whereby $x_{i}\in R^{L},$ the vectors **z, z**′ and matrix **Q** could be dense. Nonetheless, storing **Q** can still be computationally cheaper than retaining $N_{\omega_{j}}$ reference patterns **x**_*i*_ per class in main-memory – providing that $N_{\omega_{j}}>L$.

The current paper presents the mathematical aspect of our idea. Practical realizations of the proposed scheme will be given elsewhere.

## 5 Conclusion

The Parzen Window method is a powerful tool for estimating class conditional probability density functions. However, it can suffer from a severe computational bottleneck when the training dataset is large. Over the years, attempts have been made to rectify this computational drawback of the method. To the best of our knowledge the issue has remained unsolved. In this paper we have proposed a novel scheme, which we hope contributes to our endeavor of alleviating the computational bottleneck from which the Parzen Window algorithm suffers when the training dataset is large.

## Appendix A

Proposition 1 Let *a_i_* and *b_i_* be real variables ∈ [0, ∞). Then the product of $\left(\sum_{i=1}^{n}b_{i}\right){\left(\sum_{i=1}^{n}a_{i}\right)}^{q}$ can be given as $\sum_{i=1}^{n}b_{i}{\left(a_{i}\right)}^{q}+\sum_{j=1}^{q}{\left(\sum_{i=1}^{n}a_{i}\right)}^{j-1}\sum_{i=1}a_{i}\sum_{i^{\prime }\neq i}^{n}b_{i^{\prime }}{\left(a_{i^{\prime }}\right)}^{q-j},$ where $i^{\prime }=1,2,\ldots ,n$ with *n* and *q*$\in Z^{+}$.
Proof Let us suppose, with no loss of generality, that n=4 and q=3. In this scenario, we have the product $\left(b_{1}+b_{2}+b_{3}+b_{4}\right)\times \left(a_{1}+a_{2}+a_{3}+a_{4}\right)\times \left(a_{1}+a_{2}+a_{3}+a_{4}\right)\times \left(a_{1}+a_{2}+a_{3}+a_{4}\right)$. For the sake of clarity, we write the product as $\left(d_{1}+d_{2}+d_{3}+d_{4}\right)\times \left(c_{1}+c_{2}+c_{3}+c_{4}\right)\times \left(a_{1}+a_{2}+a_{3}+a_{4}\right)\times \left(b_{1}+b_{2}+b_{3}+b_{4}\right),$ where $a_{i}=c_{i}=d_{i},$ with *i* being 1, 2, 3, 4. To labour the obvious, we express $\left(d_{1}+d_{2}+d_{3}+d_{4}\right)\times \left(c_{1}+c_{2}+c_{3}+c_{4}\right)\times \left(a_{1}+a_{2}+a_{3}+a_{4}\right)\times \left(b_{1}+b_{2}+b_{3}+b_{4}\right)$ as follows

(1) $$\begin{array}{ccc} & & \left(d_{1}+d_{2}+d_{3}+d_{4}\right)\times \left(c_{1}+c_{2}+c_{3}+c_{4}\right)\\ & & \;\times \left(a_{1}+a_{2}+a_{3}+a_{4}\right)\times \left(b_{1}+b_{2}+b_{3}+b_{4}\right)\\ & & \;=b_{1}a_{1}c_{1}d_{1}+b_{2}a_{2}c_{2}d_{2}+b_{3}a_{3}c_{3}d_{3}+b_{4}a_{4}c_{4}d_{4}\\ & & \;+\;d_{1}\left(b_{2}a_{2}c_{2}+b_{3}a_{3}c_{3}+b_{4}a_{4}c_{4}\right)\\ & & \;+\;d_{2}\left(b_{1}a_{1}c_{1}+b_{3}a_{3}c_{3}+b_{4}a_{4}c_{4}\right)\\ & & \;+\;d_{3}\left(b_{1}a_{1}c_{1}+b_{2}a_{2}c_{2}+b_{4}a_{4}c_{4}\right)\\ & & \;+\;d_{4}\left(b_{1}a_{1}c_{1}+b_{2}a_{2}c_{2}+b_{3}a_{3}c_{3}\right)\\ & & \;+\;d_{1}(c_{1}\left(b_{2}a_{2}+b_{3}a_{3}+b_{4}a_{4}\right)+c_{2}\left(b_{1}a_{1}+b_{3}a_{3}+b_{4}a_{4}\right)\\ & & \;+\;c_{3}\left(b_{1}a_{1}+b_{2}a_{2}+b_{4}a_{4}\right)+c_{4}\left(b_{1}a_{1}+b_{2}a_{2}+b_{3}a_{3}\right))\\ & & \;+\;d_{2}(c_{1}\left(b_{2}a_{2}+b_{3}a_{3}+b_{4}a_{4}\right)+c_{2}\left(b_{1}a_{1}+b_{3}a_{3}+b_{4}a_{4}\right)\\ & & \;+\;c_{3}\left(b_{1}a_{1}+b_{2}a_{2}+b_{4}a_{4}\right)+c_{4}\left(b_{1}a_{1}+b_{2}a_{2}+b_{3}a_{3}\right))\\ & & \;+\;d_{3}(c_{1}\left(b_{2}a_{2}+b_{3}a_{3}+b_{4}a_{4}\right)+c_{2}\left(b_{1}a_{1}+b_{3}a_{3}+b_{4}a_{4}\right)\\ & & \;+\;c_{3}\left(b_{1}a_{1}+b_{2}a_{2}+b_{4}a_{4}\right)+c_{4}\left(b_{1}a_{1}+b_{2}a_{2}+b_{3}a_{3}\right))\\ & & \;+\;d_{4}(c_{1}\left(b_{2}a_{2}+b_{3}a_{3}+b_{4}a_{4}\right)+c_{2}\left(b_{1}a_{1}+b_{3}a_{3}+b_{4}a_{4}\right)\\ & & \;+\;c_{3}\left(b_{1}a_{1}+b_{2}a_{2}+b_{4}a_{4}\right)+c_{4}\left(b_{1}a_{1}+b_{2}a_{2}+b_{3}a_{3}\right))\\ & & \;+\;d_{1}[c_{1}(a_{1}\left(b_{2}+b_{3}+b_{4}\right)+a_{2}\left(b_{1}+b_{3}+b_{4}\right)\\ & & \;+\;a_{3}\left(b_{1}+b_{2}+b_{4}\right)+a_{4}\left(b_{1}+b_{2}+b_{3}\right))+\ldots \\ & & \;+\;c_{4}(a_{1}\left(b_{2}+b_{3}+b_{4}\right)+a_{2}\left(b_{1}+b_{3}+b_{4}\right)\\ & & \;+\;a_{3}\left(b_{1}+b_{2}+b_{4}\right)+a_{4}\left(b_{1}+b_{2}+b_{3}\right))]\\ & & \;+\;d_{2}[c_{1}(a_{1}\left(b_{2}+b_{3}+b_{4}\right)+a_{2}\left(b_{1}+b_{3}+b_{4}\right)\\ & & \;+\;a_{3}\left(b_{1}+b_{2}+b_{4}\right)+a_{4}\left(b_{1}+b_{2}+b_{3}\right))+\ldots \\ & & \;+\;c_{4}(a_{1}\left(b_{2}+b_{3}+b_{4}\right)+a_{2}\left(b_{1}+b_{3}+b_{4}\right)\\ & & \;+\;a_{3}\left(b_{1}+b_{2}+b_{4}\right)+a_{4}\left(b_{1}+b_{2}+b_{3}\right))]\\ & & \;+\;d_{3}[c_{1}(a_{1}\left(b_{2}+b_{3}+b_{4}\right)+a_{2}\left(b_{1}+b_{3}+b_{4}\right)\\ & & \;+\;a_{3}\left(b_{1}+b_{2}+b_{4}\right)+a_{4}\left(b_{1}+b_{2}+b_{3}\right))+\ldots \\ & & \;+\;c_{4}(a_{1}\left(b_{2}+b_{3}+b_{4}\right)+a_{2}\left(b_{1}+b_{3}+b_{4}\right)\\ & & \;+\;a_{3}\left(b_{1}+b_{2}+b_{4}\right)+a_{4}\left(b_{1}+b_{2}+b_{3}\right))]\\ & & \;+\;d_{4}[c_{1}(a_{1}\left(b_{2}+b_{3}+b_{4}\right)+a_{2}\left(b_{1}+b_{3}+b_{4}\right)\\ & & \;+\;a_{3}\left(b_{1}+b_{2}+b_{4}\right)+a_{4}\left(b_{1}+b_{2}+b_{3}\right))+\ldots \\ & & \;+\;c_{4}(a_{1}\left(b_{2}+b_{3}+b_{4}\right)+a_{2}\left(b_{1}+b_{3}+b_{4}\right)\\ & & \;+\;a_{3}\left(b_{1}+b_{2}+b_{4}\right)+a_{4}\left(b_{1}+b_{2}+b_{3}\right))], \end{array}$$

which can be written more compactly as:

(2) $$\begin{array}{ccc} \left(\sum_{i=1}^{4}b_{i}\right){\left(\sum_{i=1}^{n}a_{i}\right)}^{3} & = & \sum_{i=1}^{4}b_{i}{\left(a_{i}\right)}^{3}+\sum_{i=1}^{4}a_{i}\sum_{i^{\prime }\neq i}^{4}b_{i^{\prime }}{\left(a_{i^{\prime }}\right)}^{2}\\ & & +\sum_{i=1}^{4}a_{i}\sum_{i=1}^{4}a_{i}\sum_{i^{\prime }\neq i}^{4}b_{i^{\prime }}a_{i^{\prime }}\\ & & +{\left(\sum_{i=1}^{4}a_{i}\right)}^{2}\sum_{i=1}^{4}a_{i}\sum_{i^{\prime }\neq i}^{4}b_{i^{\prime }} \end{array}$$

where $i^{\prime }=1,2,3,4$; note that in Eq. 2 we have made use of fact that, by definition, $a_{i}=c_{i}=d_{i}$. It readily follows that for the general case

(3) $$\begin{array}{ccc} \left(\sum_{i=1}^{n}b_{i}\right){\left(\sum_{i=1}^{n}a_{i}\right)}^{q} & = & \sum_{i=1}^{n}b_{i}{\left(a_{i}\right)}^{q}+\sum_{i=1}^{n}a_{i}\sum_{i^{\prime }\neq i}^{n}b_{i^{\prime }}{\left(a_{i^{\prime }}\right)}^{q-1}\\ & & +\sum_{i=1}^{n}a_{i}\sum_{i=1}^{n}a_{i}\sum_{i^{\prime }\neq i}^{n}b_{i^{\prime }}{\left(a_{i^{\prime }}\right)}^{q-2}+\ldots \\ & & \ldots +{\left(\sum_{i=1}^{n}a_{i}\right)}^{q-1}\sum_{i=1}^{n}a_{i}\sum_{i^{\prime }\neq i}^{n}b_{i^{\prime }}, \end{array}$$

or in a more compact form

(4) $$\begin{array}{ccc} \left(\sum_{i=1}^{n}b_{i}\right){\left(\sum_{i=1}^{n}a_{i}\right)}^{q} & = & \sum_{i=1}^{n}b_{i}{\left(a_{i}\right)}^{q}+\sum_{j=1}^{q}{\left(\sum_{i=1}^{n}a_{i}\right)}^{j-1}\\ & & \times \sum_{i=1}a_{i}\sum_{i^{\prime }\neq i}^{n}b_{i^{\prime }}{\left(a_{i^{\prime }}\right)}^{q-j}, \end{array}$$

which can be immediately proved by induction, see Appendix B. This completes the proof of the proposition. □

Clearly Eq. 3 can be rearranged to give

(5) $$\begin{array}{ccc} \sum_{i=1}^{n}b_{i}{\left(a_{i}\right)}^{q} & = & \left(\sum_{i=1}^{n}b_{i}\right){\left(\sum_{i=1}^{n}a_{i}\right)}^{q}-\sum_{i=1}^{n}a_{i}\sum_{i^{\prime }\neq i}^{n}b_{i^{\prime }}{\left(a_{i^{\prime }}\right)}^{q-1}\\ & & -\sum_{i=1}^{n}a_{i}\sum_{i=1}^{n}a_{i}\sum_{i^{\prime }\neq i}^{n}b_{i^{\prime }}{\left(a_{i^{\prime }}\right)}^{q-2}-\ldots \\ & & \ldots -{\left(\sum_{i=1}^{n}a_{i}\right)}^{q-1}\sum_{i=1}^{n}a_{i}\sum_{i^{\prime }\neq i}^{n}b_{i^{\prime }} \end{array}$$

Remark 1 From Eqs. 1–3, one does not fail to observe that, if *n* is large (i.e. asymptotically),

(6) $$\begin{array}{ccc} {\left(\sum_{i=1}^{n}a_{i}\right)}^{q-1}\sum_{i=1}^{n}a_{i}\sum_{i^{\prime }\neq i}^{n}b_{i^{\prime }} & > & {\left(\sum_{i=1}^{n}a_{i}\right)}^{q-2}\sum_{i=1}^{n}a_{i}\sum_{i^{\prime }\neq i}^{n}b_{i^{\prime }}a_{i^{\prime }}\\ & > & {\left(\sum_{i=1}^{n}a_{i}\right)}^{q-3}\sum_{i=1}^{n}a_{i}\sum_{i^{\prime }\neq i}^{n}b_{i^{\prime }}{\left(a_{i^{\prime }}\right)}^{2}\\ & > & {\left(\sum_{i=1}^{n}a_{i}\right)}^{q-4}\sum_{i=1}^{n}a_{i}\sum_{i^{\prime }\neq i}^{n}b_{i^{\prime }}{\left(a_{i^{\prime }}\right)}^{3}\\ & > & \ldots >\sum_{i=1}^{n}a_{i}\sum_{i^{\prime }\neq i}^{n}b_{i^{\prime }}{\left(a_{i^{\prime }}\right)}^{q-1}>\sum_{i=1}^{n}b_{i}{\left(a_{i}\right)}^{q} \end{array}$$

Furthermore, a closer look at the terms above also reveals that one might – albeit asymptotically – view

$$\begin{array}{ccc} & & {\left(\sum_{i=1}^{n}a_{i}\right)}^{q-1}\sum_{i=1}^{n}a_{i}\sum_{i^{\prime }\neq i}^{n}b_{i^{\prime }},{\left(\sum_{i=1}^{n}a_{i}\right)}^{q-2}\sum_{i=1}^{n}a_{i}\sum_{i^{\prime }\neq i}^{n}b_{i^{\prime }}a_{i^{\prime }},\\ & & \ldots ,\sum_{i=1}^{n}a_{i}\sum_{i^{\prime }\neq i}^{n}b_{i^{\prime }}{\left(a_{i^{\prime }}\right)}^{q-1},\sum_{i=1}^{n}b_{i}{\left(a_{i}\right)}^{q} \end{array}$$

as a geometric progression whose common ratio is in the interval (0,1).
Remark 2 When *n* is large (that is, asymptotically), another important inference that one could glean from Eqs. 1–3 is that

(7) $$\sum_{i^{\prime }\neq 1}^{n}b_{i^{\prime }}{\left(a_{i^{\prime }}\right)}^{m}=\sum_{i^{\prime }\neq 2}^{n}b_{i^{\prime }}{\left(a_{i^{\prime }}\right)}^{m}=\sum_{i^{\prime }\neq 3}^{n}b_{i^{\prime }}{\left(a_{i^{\prime }}\right)}^{m}=\ldots =\sum_{i^{\prime }\neq n}^{n}b_{i^{\prime }}{\left(a_{i^{\prime }}\right)}^{m},$$

and even more so in our pattern recognition context whose basic credo is that all reference/prototype patterns **x**_*i*_ for a given class are similar. $i^{\prime }=1,2,\ldots ,n$ and m=q−l, where l=1,2,…,q−1

Proposition 1 , Remarks 1 and 2 essentially form the theoretical basis for the algorithm proposed in this work.

Based on Remark 1, it is justifiable to assume that Eq. 5 can be well approximated as

(8) $$\begin{array}{ccc} \sum_{i=1}^{n}b_{i}{\left(a_{i}\right)}^{q} & \approx & \left(\sum_{i=1}^{n}b_{i}\right){\left(\sum_{i=1}^{n}a_{i}\right)}^{q}-{\left(\sum_{i=1}^{n}a_{i}\right)}^{q-1}\sum_{i=1}^{n}a_{i}\sum_{i^{\prime }\neq i}^{n}b_{i^{\prime }}\\ & & -{\left(\sum_{i=1}^{n}a_{i}\right)}^{q-2}\sum_{i=1}^{n}a_{i}\sum_{i^{\prime }\neq i}^{n}b_{i^{\prime }}a_{i^{\prime }} \end{array}$$

Due to space constraints, practical realization and validation of Eq. 8 will be given in application journals on pattern recognition and cheminformatics.

## Appendix B

Proof of Eq. 4 by induction.

*Base case*: when q=1, from Eq. 4 we have

(9) $$\begin{array}{ccc} \left(\sum_{i=1}^{n}b_{i}\right){\left(\sum_{i=1}^{n}a_{i}\right)}^{1} & = & \sum_{i=1}^{n}b_{i}{\left(a_{i}\right)}^{1}\\ & & +\sum_{j=1}^{1}{\left(\sum_{i=1}^{n}a_{i}\right)}^{j-1}\sum_{i=1}^{n}a_{i}\sum_{i^{\prime }\neq i}^{n}b_{i^{\prime }}{\left(a_{i^{\prime }}\right)}^{1-j} \end{array}$$

Clearly the left hand side of the equation can be expressed as $\sum_{i=1}^{n}b_{i}a_{i}+\sum_{i=1}^{n}a_{i}\sum_{i^{\prime }\neq i}^{n}b_{i^{\prime }}$ and the right hand side of the equation is $\sum_{i=1}^{n}b_{i}a_{i}+\sum_{i}a_{i}\sum_{i^{\prime }\neq i}^{n}b_{i^{\prime }}$. Thus Eq. 9 holds for q=1.

*General case:*

Assume that Eq. 4 is correct for some positive integer $k\in Z^{+}$. From Eq. 4 we have

(10) $$\begin{array}{ccc} \left(\sum_{i=1}^{n}b_{i}\right){\left(\sum_{i=1}^{n}a_{i}\right)}^{k} & = & \sum_{i=1}^{n}b_{i}{\left(a_{i}\right)}^{k}\\ & & +\sum_{j=1}^{k}{\left(\sum_{i=1}^{n}a_{i}\right)}^{j-1}\sum_{i}a_{i}\sum_{i^{\prime }\neq i}^{n}b_{i^{\prime }}{\left(a_{i^{\prime }}\right)}^{k-j} \end{array}$$

We now show that the equation above holds also for k+1. Starting with the left hand side, we have

(11) $$\begin{array}{ccc} \left(\sum_{i=1}^{n}b_{i}\right){\left(\sum_{i=1}^{n}a_{i}\right)}^{k}\left(\sum_{i=1}^{n}a_{i}\right) & = & (\sum_{i=1}^{n}b_{i}{\left(a_{i}\right)}^{k}+\sum_{j=1}^{k}{\left(\sum_{i=1}^{n}a_{i}\right)}^{j-1}\\ & & \times \sum_{i=1}^{n}a_{i}\sum_{i^{\prime }\neq i}^{n}b_{i^{\prime }}{\left(a_{i^{\prime }}\right)}^{k-j})\left(\sum_{i=1}^{n}a_{i}\right), \end{array}$$

where we have made use of the assumption that Eq. 10 is correct; hence

(12) $$\begin{array}{ccc} \left(\sum_{i=1}^{n}b_{i}\right){\left(\sum_{i=1}^{n}a_{i}\right)}^{k}\left(\sum_{i=1}^{n}a_{i}\right) & = & \sum_{i=1}^{n}b_{i}{\left(a_{i}\right)}^{k}\left(\sum_{i=1}^{n}a_{i}\right)+\sum_{j=1}^{k}\\ & & \times {\left(\sum_{i=1}^{n}a_{i}\right)}^{j}\sum_{i}a_{i}\sum_{i^{\prime }\neq i}^{n}b_{i^{\prime }}{\left(a_{i^{\prime }}\right)}^{k-j}, \end{array}$$

where $\sum_{i=1}^{n}b_{i}{\left(a_{i}\right)}^{k}\left(\sum_{i=1}^{n}a_{i}\right)$ can be expressed as

(13) $$\sum_{i=1}^{n}b_{i}{\left(a_{i}\right)}^{k}\left(\sum_{i=1}^{n}a_{i}\right)=\sum_{i=1}^{n}b_{i}{\left(a_{i}\right)}^{k+1}+\sum_{i=1}^{n}a_{i}\sum_{i^{\prime }\neq i}^{n}b_{i^{\prime }}{\left(a_{i^{\prime }}\right)}^{k}$$

Inserting Eq. 13 into Eq. 12 results in

(14) $$\begin{array}{ccc} & & \left(\sum_{i=1}^{n}b_{i}\right){\left(\sum_{i=1}^{n}a_{i}\right)}^{k}\left(\sum_{i=1}^{n}a_{i}\right)=\sum_{i=1}^{n}b_{i}{\left(a_{i}\right)}^{k+1}\\ & & \;+\left[\sum_{i=1}^{n}a_{i}\sum_{i^{\prime }\neq i}^{n}b_{i^{\prime }}{\left(a_{i^{\prime }}\right)}^{k}+\sum_{j=1}^{k}{\left(\sum_{i=1}^{n}a_{i}\right)}^{j}\sum_{i}a_{i}\sum_{i^{\prime }\neq i}^{n}b_{i^{\prime }}{\left(a_{i^{\prime }}\right)}^{k-j}\right] \end{array}$$

$\left[\sum_{i=1}^{n}a_{i}\sum_{i^{\prime }\neq i}^{n}b_{i^{\prime }}{\left(a_{i^{\prime }}\right)}^{k}+\sum_{j=1}^{k}{\left(\sum_{i=1}^{n}a_{i}\right)}^{j}\sum_{i}a_{i}\sum_{i^{\prime }\neq i}^{n}b_{i^{\prime }}{\left(a_{i^{\prime }}\right)}^{k-j}\right]$ is basically $\sum_{j=0}^{k}{\left(\sum_{i=1}^{n}a_{i}\right)}^{j}\sum_{i}a_{i}\sum_{i^{\prime }\neq i}^{n}b_{i^{\prime }}{\left(a_{i^{\prime }}\right)}^{k-j},$ which can be rewritten as $\sum_{j=1}^{k+1}{\left(\sum_{i=1}^{n}a_{i}\right)}^{j-1}\sum_{i=1}a_{i}\sum_{i^{\prime }\neq i}^{n}b_{i^{\prime }}{\left(a_{i^{\prime }}\right)}^{(k+1)-j}$. Inserting this expression in Eq. 14 and rewriting ${\left(\sum_{i=1}^{n}a_{i}\right)}^{k}\left(\sum_{i=1}^{n}a_{i}\right)$ on the left hand side of Eq. 14 as ${\left(\sum_{i=1}^{n}a_{i}\right)}^{k+1}$ yield

(15) $$\begin{array}{ccc} & & \left(\sum_{i=1}^{n}b_{i}\right){\left(\sum_{i=1}^{n}a_{i}\right)}^{k+1}=\sum_{i=1}^{n}b_{i}{\left(a_{i}\right)}^{k+1}\\ & & \;+\left[\sum_{j=1}^{k+1}{\left(\sum_{i=1}^{n}a_{i}\right)}^{j-1}\sum_{i=1}a_{i}\sum_{i^{\prime }\neq i}^{n}b_{i^{\prime }}{\left(a_{i^{\prime }}\right)}^{(k+1)-j}\right], \end{array}$$

which is what we set out to prove. Thus, Eq. 4 (and for that matter Eq. 3) in Appendix A hold for all values of $q\in Z^{+}$.

## Appendix C

In this work: $b_{i}=x^{T}\left(\beta_{i}x_{i}\right)$; $b_{i^{\prime }}=x^{T}\left(\beta_{i^{\prime }}x_{i^{\prime }}\right)$; $a_{i}=x^{T}x_{i}$; $a_{i^{\prime }}=x^{T}x_{i^{\prime }}$; *q* = *r - 1*; and *n* = $N_{\omega_{j}},$ where **x**, *β_i_*, $\beta_{i^{\prime }},$ **x**_*i*_, **x**${}_{i^{\prime }},$ *r* and $N_{\omega_{j}}$ are as described in the main text (see Section 3). Hence, from Eq. 8 we have

(16) $$\begin{array}{ccc} \sum_{i=1}^{N_{\omega_{j}}}\left(x^{T}\left(\beta_{i}x_{i}\right)\right){\left(x^{T}x_{i}\right)}^{r-1} & \approx & c^{r-1}\left(x^{T}\sum_{i=1}^{N_{\omega_{j}}}\left(\beta_{i}x_{i}\right)\right)\\ & & -c^{r-2}\left(\sum_{i=1}^{N_{\omega_{j}}}x^{T}x_{i}\sum_{i\neq i^{\prime }}^{N_{\omega_{j}}}x^{T}\left(\beta_{i^{\prime }}x_{i^{\prime }}\right)\right)\\ & & -c^{r-3}\left(\sum_{i=1}^{N_{\omega_{j}}}x^{T}x_{i}\sum_{i\neq i^{\prime }}^{N_{\omega_{j}}}\left(x^{T}\left(\beta_{i^{\prime }}x_{i^{\prime }}\right)\right)\left(x^{T}x_{i^{\prime }}\right)\right) \end{array}$$

where $c=(x^{T}\sum_{i=1}^{N_{\omega_{j}}}x_{i})$.

Making use of the two definitions $z^{\prime }=\sum_{i=1}^{N_{\omega_{j}}}\left(\beta_{i}x_{i}\right)$ and $z=\sum_{i=1}^{N_{\omega_{j}}}x_{i}$ given in Section 3 in the main text and with some algebraic manipulation (such as the fact that **g**^*T*^**h** = **h**^*T*^**b** for any vectors **g** and **h**), the three lines on the right hand side of Eq. 16 can be recast in a more simple and familiar form:

• Line 1: $c^{r-1}\left(x^{T}\sum_{i=1}^{N_{\omega_{j}}}\left(\beta_{i}x_{i}\right)\right)={\left(x^{T}z\right)}^{r-1}x^{T}z^{\prime }$
• Line 2: $c^{r-2}={\left(x^{T}z\right)}^{r-2},$ whereas
  $$\left(\sum_{i=1}^{N_{\omega_{j}}}x^{T}x_{i}\sum_{i\neq i^{\prime }}^{N_{\omega_{j}}}x^{T}\left(\beta_{i^{\prime }}x_{i^{\prime }}\right)\right)=x^{T}Yx,$$
  where $Y=(\sum_{i=1}^{N_{\omega_{j}}}x_{i}\sum_{i\neq i^{\prime }}^{N_{\omega_{j}}}{\left(\beta_{i^{\prime }}x_{i^{\prime }}\right)}^{T})$ is an *L*-by-*L* matrix.
• Line 3: $c^{r-3}={\left(x^{T}z\right)}^{r-3},$ whereas
  (17) $$\begin{array}{ccc} & & \left(\sum_{i=1}^{N_{\omega_{j}}}x^{T}x_{i}\sum_{i\neq i^{\prime }}^{N_{\omega_{j}}}\left(x^{T}\left(\beta_{i^{\prime }}x_{i^{\prime }}\right)\right)\left(x^{T}x_{i^{\prime }}\right)\right)\\ & & \;=x^{T}\sum_{i=1}^{N_{\omega_{j}}}x_{i}x^{T}\sum_{i\neq i^{\prime }}^{N_{\omega_{j}}}\left(\left(\beta_{i^{\prime }}x_{i^{\prime }}\right)x_{i^{\prime }}^{T}\right)x\\ & & \;=x^{T}\sum_{i=1}^{N_{\omega_{j}}}x_{i}\left(x^{T}S_{i}x\right), \end{array}$$
  where $S_{i}=\sum_{i\neq i^{\prime }}^{N_{\omega_{j}}}\left(\left(\beta_{i^{\prime }}x_{i^{\prime }}\right)x_{i^{\prime }}^{T}\right)$ is an *L*-by-*L* matrix.
  According to Eq. 7, the matrices $S_{1},S_{2},\ldots ,S_{N_{\omega_{j}}}$ can be considered equivalent/similar. Hence, Eq. 17 modifies to
  (18) $$\left(\sum_{i=1}^{N_{\omega_{j}}}x^{T}x_{i}\sum_{i\neq i^{\prime }}^{N_{\omega_{j}}}\left(x^{T}\left(\beta_{i^{\prime }}x_{i^{\prime }}\right)\right)\left(x^{T}x_{i^{\prime }}\right)\right)=\left(x^{T}z\right)\left(x^{T}Sx\right),$$
  where **S** = **S**_*i*_ and $\left(x^{T}z\right)=x^{T}\sum_{i=1}^{N_{\omega_{j}}}x_{i}$

Expressed in terms of **Y, S, z**′ and **z**, Eq. 16 becomes

(19) $$\begin{array}{ccc} & & \sum_{i=1}^{N_{\omega_{j}}}\left(x^{T}\left(\beta_{i}x_{i}\right)\right){\left(x^{T}x_{i}\right)}^{r-1}\\ & & \;\approx {\left(x^{T}z\right)}^{r-1}\left(x^{T}z^{\prime }\right)-{\left(x^{T}z\right)}^{r-2}\left(x^{T}\left(Y+S\right)x\right)\\ & & \;={\left(x^{T}z\right)}^{r-2}\left(\left(x^{T}z^{\prime }\right)\left(x^{T}z\right)-x^{T}\left(Y+S\right)x\right)\\ & & \;={\left(x^{T}z\right)}^{r-2}\left(\left(x^{T}z^{\prime }z^{T}x\right)-x^{T}\left(Y+S\right)x\right)\\ & & \;={\left(x^{T}z\right)}^{r-2}\left(x^{T}Qx\right), \end{array}$$

where $Q=z^{\prime }z^{T}-\left(Y+S\right),$ an *L*-by-*L* matrix.

Note that ${\left(x^{T}z\right)}^{r-2}\geq 0,$$\left(x^{T}z^{\prime }z^{T}x\right)\geq x^{T}\left(Y+S\right)x$; hence, ${\left(x^{T}z\right)}^{r-2}\left(x^{T}Qx\right)\geq 0$ as it should be – because, by definition, $\sum_{i=1}^{N_{\omega_{j}}}\left(x^{T}\left(\beta_{i}x_{i}\right)\right){\left(x^{T}x_{i}\right)}^{r-1}\geq 0$.
